# Quasi-experimental evaluation of Kenya’s pictorial health warnings versus Zambia’s single text-only warning: findings from the International Tobacco Control (ITC) Project

**DOI:** 10.1136/tobaccocontrol-2020-056396

**Published:** 2021-06-11

**Authors:** Susan Cherop Kaai, Genevieve Sansone, Gang Meng, Jane Rahedi Ong’ang’o, Fastone Goma, Lawrence Ikamari, Anne C K Quah, Geoffrey T Fong

**Affiliations:** 1 Department of Psychology, University of Waterloo, Waterloo, Ontario, Canada; 2 School of Public Health and Health Systems, University of Waterloo, Waterloo, Ontario, Canada; 3 Faculty of Social Work, University of Toronto, Toronto, Ontario, Canada; 4 Centre for Respiratory Disease Research, Kenya Medical Research Institute, Nairobi, Kenya; 5 School of Medicine, University of Zambia, Lusaka, Zambia; 6 Centre for Primary Care Research, Eden University, Lusaka, Zambia; 7 Population Studies and Research Institute, University of Nairobi, Nairobi, Kenya; 8 Ontario Institute for Cancer Research, Toronto, Ontario, Canada

**Keywords:** global health, packaging and labelling, public policy, low/middle income country

## Abstract

**Background:**

Population studies in mostly high-income countries have shown that pictorial health warnings (PHWs) are much more effective than text-only warnings. This is the first quasi-experimental evaluation of the introduction of PHWs in Africa, comparing the change from text-only to PHWs in Kenya to the unchanged text-only health warning in Zambia.

**Methods:**

Data were from International Tobacco Control (ITC) Surveys in Kenya (n=1495), and Zambia (n=1628), cohort surveys of nationally representative samples of adult smokers in each country. The ITC Kenya Survey was conducted in 2012 and 2018 (2 years after the 2016 introduction of three PHWs). The ITC Zambia Survey was conducted in 2012 and 2014 with no change to the single text-only warning. Validated indicators of health warning effectiveness (HWIs) (salience: noticing, reading; cognitive reactions: thinking about health risks, thinking about quitting; and behavioural reactions: avoiding warnings; forgoing a cigarette because of the warnings), and a summary measure—the Labels Impact Index (LII)—measured changes in warning impact between the two countries.

**Results:**

PHWs implemented in Kenya led to a significant increase in all HWIs and the LII, compared with the text-only warning in Zambia. The failure to implement PHWs in Zambia led to a substantial missed opportunity to increase warning effectiveness (eg, an estimated additional 168 392 smokers in Zambia would have noticed the warnings).

**Conclusions:**

The introduction of PHWs in Kenya substantially increased the effectiveness of warnings. These results provide strong empirical support for 34 African countries that still have text-only warnings, of which 31 are Parties of the Framework Convention on Tobacco Control and are thus obligated to implement PHWs.

## Introduction

Tobacco smoking kills 7.1 million smokers annually, with an additional 1.2 million non-smokers dying from secondhand smoke.^1^ About 77 million adult smokers reside in Africa, and this figure is projected to increase to 413 million by 2100 if current trends persist in the absence of effective tobacco control interventions.^2^ Among the policy interventions of the WHO Framework Convention on Tobacco Control (FCTC), the first WHO treaty,^3^ are pictorial health warnings (PHWs) on all tobacco product packaging and labelling that cover 50% or more of the principal display areas.^4^ As of February 2021, 126 countries and jurisdictions have required PHWs.^5^

Health warnings on tobacco packaging are among the most direct and cost-effective means of communicating the health risks of tobacco use to the public.^4^ A pack-a-day smoker is potentially exposed to warnings about 7300 times per year (20 views /day × 365 days/year).^6^ There are probably no other interventions in health that are delivered appropriately and so often, and its cost-effectiveness makes health warnings especially important in low-income and middle-income countries (LMICs), where 80% of tobacco-related deaths are projected to occur this century.^7^

It is well established that PHWs are more effective than text-only warnings in communicating health risks of tobacco use. PHWs are more likely to: be noticed or read^8–17^; better communicate the health risks of tobacco use^18–20^; increase thoughts about the health risks of tobacco use and about quitting^8 11 13 21^; be rated as more effective by tobacco users^12 22 23^; encourage tobacco users to forgo a cigarette or avoid looking at the warning^8 11^; increase motivation and intention to quit^24^; increase cessation among adult smokers^25 26^; be associated with more quit attempts among youth smokers and decreased uptake of tobacco use among non-smoking youth^12^ and decrease adult smoking prevalence.^27^ However, nearly all evidence regarding the effectiveness of PHWs has come from studies in high-income countries (HICs); few studies have been conducted in LMICs, where the advantages of graphical warnings may be even greater, due to lower literacy levels which make text-only warnings less effective.^4 15^

The need for evidence about effectiveness of PHWs is especially high in the African Region, where there are weaker systems of tobacco control and law enforcement, very low levels of literacy, as well as low awareness of the harms of using tobacco.^28 29^ Additionally, due to rising population and significant economic growth in Africa, the tobacco industry has increased its efforts through marketing and aggressive lobbying in an effort to increase smoking in many African countries.^2 30^

Although studies from HICs have shown that PHWs are more effective in educating low literate populations on the harms of tobacco use than text-only warnings, only 13 (Burkina Faso, Cameroon, Chad, Ethiopia, Gabon, Ghana, Kenya, Madagascar, Mauritius, Namibia, Senegal, Seychelles and Togo) out of 47 countries from the African Region have successfully implemented PHWs.

There is a paucity of research on PHWs in the African Region. A cross-sectional study among adolescents from Nigeria concluded that PHWs, especially with images depicting cancer and impotence, may encourage abstinence among non-smokers.^31^ A qualitative study from Ghana found that PHWs were perceived to be more effective by both smokers and non-smokers—especially given the low literacy rates of smokers in the country.^32^

The International Tobacco Control (ITC) Project conducted a pre–post evaluation of the October 2009 introduction of PHWs among a nationally representative cohort of adult smokers in Mauritius,^33^ showing that the introduction of PHWs on cigarette packs significantly enhanced all indicators of warning effectiveness, but showed a decline in PHW effectiveness from Wave 2 to Wave 3 (a period in which PHWs remained unchanged), indicating wear out of the warnings, pointing to the need for periodic revision.^34^

**Figure 1:**
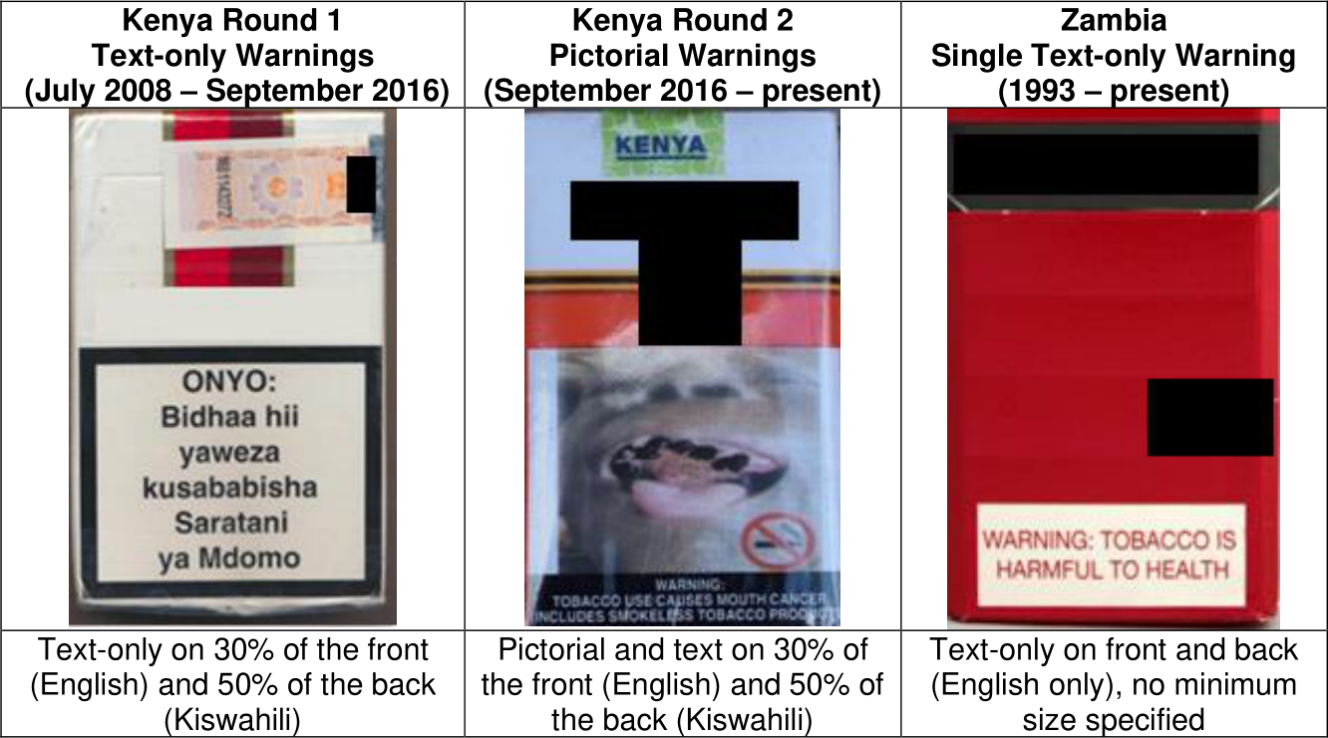
Health warnings in Kenya and Zambia.

In Kenya, health warnings were introduced as part of the 2007 Tobacco Control Act, which came into force in July 2008 and included requirements for 13 rotating text-only health warnings (30% front and 50% back of cigarette packages) in both Kiswahili and English (figure 1—round 1 Kenya text-only warnings). In 2014, the government introduced the Tobacco Control Regulations which included 15 new PHWs (30% front and 50% back) of smoked and smokeless tobacco packages. The regulations were to be implemented in June 2015 but were delayed due to a legal challenge by British American Tobacco-Kenya (BAT-K). Even though BAT-K lost their case, only three (figure 1—round 2 Kenya PHWs) out of the 15 PHWs have been implemented on cigarette packs thus far, starting in September 2016.^35^

In contrast to Kenya’s introduction of PHWs, Zambia has since 1993 required only a single text-only English health warning that occupies less than 30% at the bottom of the front and back of the pack. (figure 1—Zambia single text-only warning). While further amendments to the law were implemented in 2008 requiring strengthening the legibility of the warning, there has been no change to the actual health warning to date.^36^

The ITC Project established nationally representative cohort studies of smokers in both Kenya and Zambia, providing a unique opportunity to conduct a quasi-experimental evaluation of the September 2016 implementation of PHWs in Kenya by comparing smokers’ perceptions and behaviours before and after the implementation of the PHWs versus the single text-only warning of Zambia, which did not change.

## Method

### Study design

The ITC Kenya and Zambia Surveys are part of the larger ITC Project,^37^ which has conducted parallel longitudinal cohort surveys in 29 countries to evaluate FCTC policies. For this study, we used the evaluation conceptual framework for the ITC Project which allows for quasi‐experiments, that is, where a country that does not implement a given new tobacco control policy acts as the control group (ITC Zambia) to which the country implementing such a policy (PHWs in ITC Kenya) will be compared. The ITC Kenya Wave 1 (W1) Survey was conducted in October–December 2012 and Wave 2 (W2) was conducted in April–June 2018, approximately 2 years after the implementation of the three PHWs in September 2016. The ITC Zambia W1 Survey was conducted in September–December 2012 and W2 was conducted in August–October 2014. There was no change to the single text-only English health warning between the two waves in Zambia. Thus, Zambia was a ‘control’ country against which the introduction of PHWs in Kenya was evaluated in a quasi-experimental design.

### Participants and survey procedures

The ITC Kenya and Zambia surveys were conducted face to face among nationally representative cohorts of adult (≥18 years) tobacco users and non-users. Recruitment into the cohort followed a multistage clustered sampling design. A detailed description of the sampling and study design is available in technical reports.^35 36 38^ In Kenya, questionnaires were administered to smokers (n=1495) either in Kiswahili (national language) or English (official language), while in Zambia questionnaires were administered to smokers (n=1628) in English (official language) and five national languages: Bemba, Nyanja, Kaonde, Tonga and Lozi.

The overall retention rates in Kenya and Zambia were 44% and 64%, respectively. The average survey completion time was 60 min in both countries.

### Measures

#### Health warning label effectiveness

Warning label effectiveness was measured using three groups of validated indicators (i.e., *s*alience, cognitive reactions and behavioural reactions), which have been used to evaluate the effectiveness of health warnings in ITC studies across multiple countries.^39^ Each indicator was analysed as a dichotomous measure to estimate how frequently smokers noticed, read and had specific cognitive and behavioural reactions to the warnings in the last month (except for cognitive reactions, which did not specify a time frame).

Warning salience was measured by two questions: (1) NOTICING: ‘In the last month, how often, if at all, have you noticed warnings on cigarette packages?’ (dichotomised as: often/very often vs never or once in a while) and (2) READING: ‘In the last month, how often, if at all, have you read or looked closely at the warning labels on cigarette packages?’ (dichotomised as: often/regularly vs never/rarely/once in a while).

Cognitive reactions were measured by two questions: (1) THOUGHTS: ‘To what extent, if at all, do the warning labels on cigarette packages make you think about the health risks of smoking?’ (dichotomised as: a lot vs not at all/a little) and (2) QUITTING LIKELIHOOD: ‘To what extent, if at all, do the warning labels make you more likely to quit smoking?’ (dichotomised as: a lot vs not at all/a little).

Behavioural reactions were measured by two questions: (1) AVOIDING: ‘In the last month have you made any effort to avoid looking at or thinking about the warning labels, such as covering them up, keeping them out of sight, using a cigarette case, avoiding certain warnings, or any other means?’ (yes vs no). It should be noted that avoiding warnings has been found to be a significant predictor of future quit behaviours^24 40–42^ and (2) FORGOING: ‘In the last month, have the warning labels stopped you from having a cigarette when you were about to smoke one?’ (dichotomised as: once in a while/many times vs never).

#### Labels Impact Index

The Labels Impact Index (LII) is a composite measure that combines four of the six health warning validated indicators (NOTICING, THOUGHTS, QUITTING and FORGOING). LII was created by standardising the original measures and then weighting and summing the standardised scores as follows: LII= (NOTICING*1) + (THOUGHTS*2) + (QUITTING*2) + (FORGOING*3). Higher scores indicate greater impact.^34 43^

Demographic measures were gender (male, female) and age group (18–24, 25–39, 40–54 and 55 and older). Respondents answered questions that allowed them to be classified as either daily or non-daily smokers.

### Time-in-sample

A time-in-sample variable was constructed to represent the number of times a respondent participated in the survey to account for potential differences in individuals’ responses between those who were newly recruited compared with those who completed one prior survey wave.

### Analyses

Generalised estimating equation (GEE) regression models^44^ were used to test the within-country changes in health warning measures over time and cross-country differences in warning label effects and trends. Logistic GEE regression models were conducted for binary outcomes, and linear GEE regression models were used to test the changes in LII over time. A country × wave interaction term was included to test if the differences in health warning measures between the two survey waves were different between Kenya and Zambia. This quasi-experimental difference-in-difference (DID) estimation provided the specific test of whether the introduction of PHWs in Kenya increased the effectiveness of the warnings between the two survey waves, compared with Zambia, whose text-only warning remained unchanged between their two survey waves.

GEE models account for within-subject correlation arising when outcomes are measured on the same respondent more than once.^44^ Survey design information including strata and the primary sampling units were incorporated taking into account the complex survey design. All proportion estimates were weighted to ensure that they were representative of the population of smokers in Kenya and Zambia. Regression models controlled for time-varying (smoking status), time invariant (sex and age group) covariates and time-in-sample to ensure that any changes in the effectiveness measures were not attributable to confounding effects from these covariates. All models were estimated using SAS (V.9.2) callable SUDAAN V.11.

## Results

### Sample characteristics

Sample characteristics for the ITC Kenya and Zambia Surveys at recruitment wave are presented in table 1. In accordance with national smoking prevalence trends, most smokers in the survey samples were male (92.3% in Kenya and 95.3% in Zambia), and were daily smokers (91.6% in Kenya and 87.9% in Zambia). The mean age of the respondents was 41.1 years (SD 14.7) in Kenya and 38.9 years (SD 15.2) in Zambia.

**Table 1:** Sample characteristics at recruitment in Kenya and Zambia

Characteristics	Kenya (n=1495)		Zambia (n=1628)	
	Frequency	Per cent	Frequency	Per cent
Sex				
female	115	7.7	76	4.7
male	1380	92.3	1552	95.3
Age group				
18–24	135	9.0	265	16.3
25–39	655	43.9	712	43.7
40–54	425	28.4	393	24.1
55–max	280	18.7	258	15.9
Mean (SD)	41.1 (14.7)		38.9 (15.2)	
Smoking status				
Daily	1369	91.6	1431	87.9
Non-daily	126	8.4	197	12.1
Cohort				
1	1049	70.1	1188	73.0
2	446	29.9	440	27.0
Cohort 1 smoker retention				
Lost or quit smoking	692	66.0	583	49.1
Retained as smoker	357	34.0	605	50.9

These statistics are unweighted.

### Impact of warnings in Kenya versus Zambia

**Figure 2:**
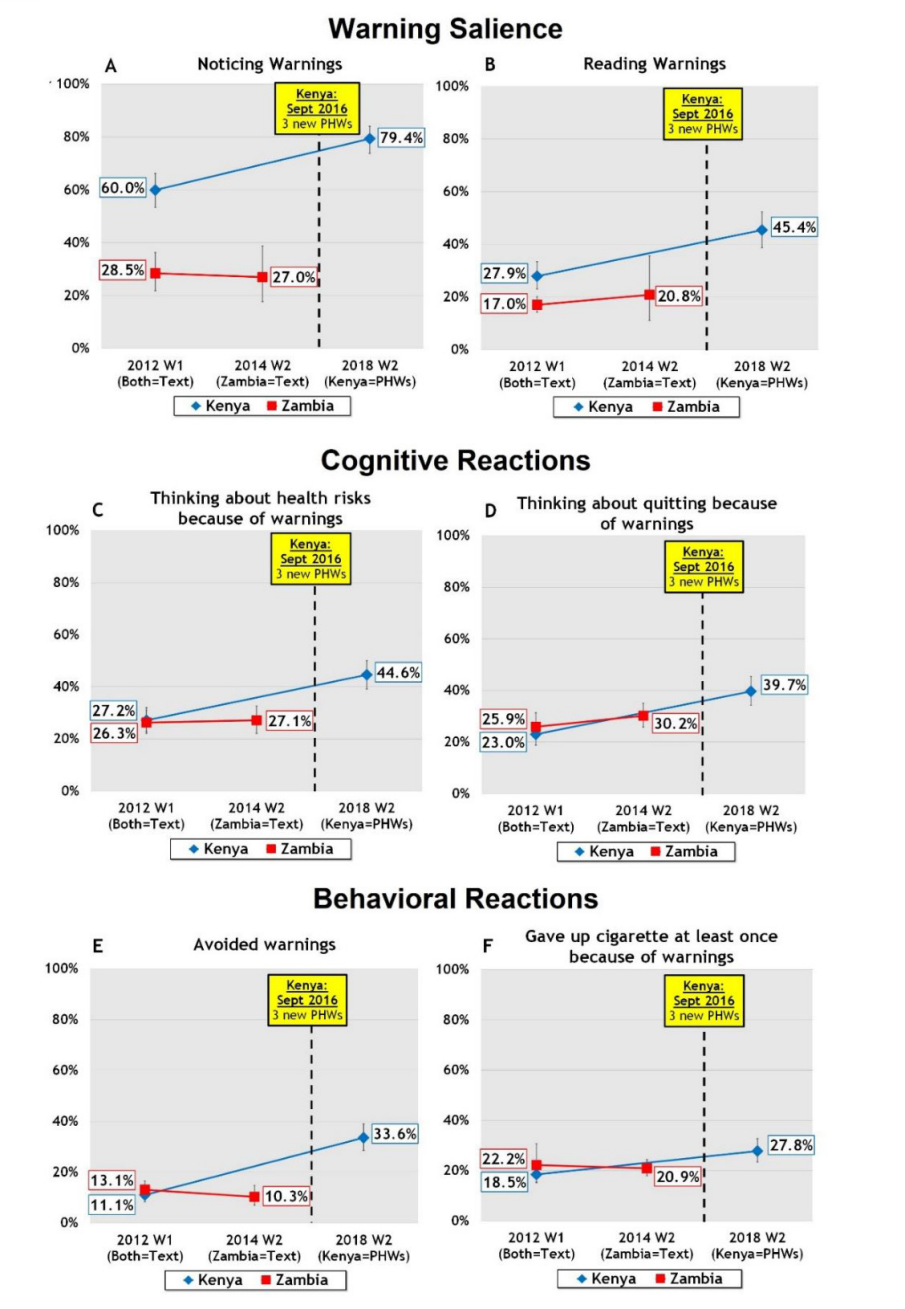
(A–F) Changes in warning salience (noticing, reading), cognitive reactions (thinking about health risks, thinking about quitting) and behavioural reactions (avoiding warnings, forgoing a cigarette) in Kenya and Zambia. PHWs, pictorial health warnings.

Each of the six indicators of health warning effectiveness (HWI) at W1 and W2 in Kenya and Zambia are presented in figure 2A–F. W1 was conducted when Kenya had 13 text-only health warnings on 30% of the front (in English) and 50% of the back of tobacco packs (in Kiswahili), while Zambia had a single text-only warning in English that occupied less than 30% on the bottom front and back of the pack (figure 1). W2 in Kenya was conducted 2 years after the implementation of three out of 15 PHWs on tobacco packs (the remaining 12 PHWs have not yet been implemented to date), and W2 in Zambia was conducted 2 years after W1, a period when the single text-only English warning did not change.

#### Warning Salience

Figure 2A, B shows that the implementation of the three PHWs in Kenya led to significant changes in both measures of warning salience between W1 and 2. After the introduction of PHWs, Kenyan smokers were more likely to notice the warnings ‘often/very often’: from 60.0% to 79.4% (adjusted OR (aOR)=2.69, 95% CI=1.74 to 4.17, p<0.001) (figure 2A) and more likely to report reading warnings closely: from 27.9% to 45.4% (aOR=2.18, 95% CI=1.41 to 3.36, p=0.0003) (figure 2B). In Zambia, there were no significant changes in the percentage of smokers who reported noticing their text health warnings (from 28.5% to 27.0%; p=0.64) or reading the warnings (from 17.0% to 20.8%; p=0.51). The quasi-experimental tests comparing the change in Kenya versus the change in Zambia were significant for both measures: noticing (DID (% change in KE - % change in ZM*)*=20.9%, p<0.001) and reading (DID=13.7%, p=0.02).

#### Cognitive reactions

Figure 2C, D shows that the implementation of PHWs in Kenya led to increases in both cognitive reactions to the warnings. Smokers were more likely to report that the warnings had made them think about smoking-related health risks: from 27.2% to 44.6% (aOR=2.17, 95% CI=1.54 to 3.06, p<0.001) (figure 2C) and to report thinking about quitting because of the warnings: from 23.0% to 39.7% (aOR=2.22, 95% CI=1.52 to 3.23, p<0.001) (figure 2D). In Zambia, there were no significant changes in the percentage of smokers who reported thinking about smoking-related health risks (from 26.3% to 27.1%; p=0.82) or thinking about quitting (from 25.9% to 30.2%; p=0.12) because of the warnings. The quasi-experimental tests comparing the change in Kenya versus the change in Zambia were significant for both cognitive indicators: thinking about risks (DID=16.6%, p<0.001) and thinking about quitting (DID=12.4%, p=0.006).

#### Behavioural reactions

Figure 2E, F shows that the implementation of PHWs in Kenya led to increases in both behavioural reactions to the warnings. Kenyan smokers were more likely to report avoiding the warnings: from 11.1% to 33.6% (aOR=4.11, 95% CI=2.71 to 6.23, p<0.001) (figure 2E) and forgoing a cigarette due to the warnings: from 18.5% to 27.8% (aOR=1.71, 95% CI=1.23 to 2.37, p=0.001) (figure 2F). In Zambia, there was no significant change in avoiding warnings in Zambia (from 13.1% to 10.3%; p=0.23) and in forgoing a cigarette because of the warnings (from 22.2% to 20.9%; p=0.68). The quasi-experimental tests comparing the change in Kenya vs the change in Zambia were significant for both behavioural indicators: avoiding warnings (DID=25.3%, p<0.001) and forgoing a cigarette (DID=10.6%, p=0.02).

#### Labels Impact Index

**Figure 3:**
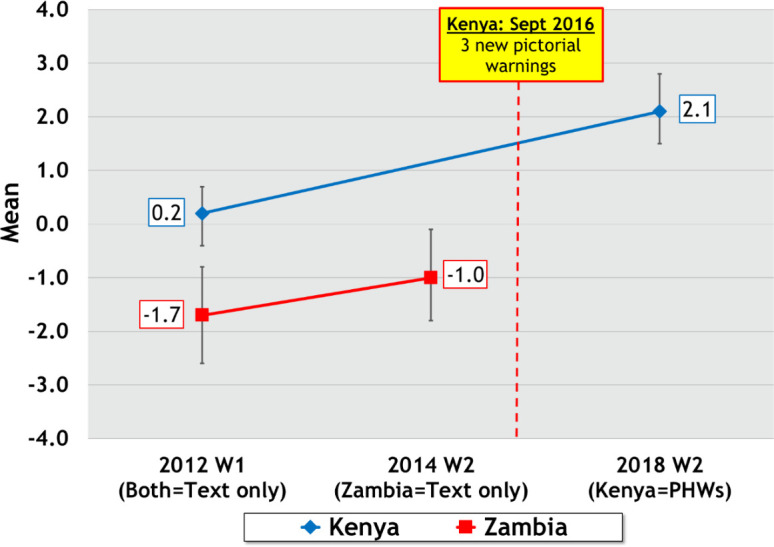
Changes in the Labels Impact Index for Kenya and Zambia. PHWs, pictorial health warnings.

The changes in the LII over the two waves in both countries are presented in figure 3. In Kenya, there was a significant positive mean change of the LII (β=1.98, p<0.001), from a mean of 0.2 at Wave 1 to 2.1 at Wave 2. There was a small significant mean change in the LII (β=0.70, p=0.03) in Zambia from W1 and W2 (ie, from a mean of −1.7 to −1.0). The country × wave interaction (the quasi-experimental test) was significant (p=0.02), showing that the LII increase in Kenya was greater than the LII increase in Zambia.

## Discussion

This is the first quasi-experimental evaluation of the introduction of PHWs in the African Region. The introduction of PHWs in Kenya in 2016 led to substantial increases in all six of the key indicators of warning effectiveness and in the overall LII compared with the text-only warnings in Zambia.

These findings are consistent with other longitudinal evaluation studies conducted in other countries that changed from text-only to PHWs, including Australia,^8^ Canada,^9 45^ The Netherlands,^46^ Malaysia,^21^ Mauritius^33^ and Thailand.^17^ This study shows that the benefits of switching to PHWs (eg, increasing awareness of harms of tobacco use among smokers) are not limited to high-income Western countries but are also applicable to LMICs such as those in the African Region. Moreover, the low impact of Zambia’s single text-only warning is consistent with ITC evidence from other countries with similar text-only warnings, such as Japan and China.^9 47^ The persistence of text-only warnings in Africa is particularly alarming given the high proportion of the population of many African countries who are not be able to read text-only warnings.^15 48^ Textual health warnings (unlike PHWs) create potential health inequalities for countries with low literacy rates.

We have cast these findings with respect to the positive impact of the Kenya pictorial warnings. An advantage of our quasi-experimental evaluation is that it also highlights the continued negative impact of the Zambia single text-only warning. For Zambia to not implement pictorial warnings, despite being an FCTC Party, constitutes a cost—a missed opportunity—which led to smokers failing to benefit from the positive impact of PHWs.

To estimate the magnitude of this missed opportunity, we conducted calculations using a method from a previous ITC study that compared China’s text-only warnings to Malaysia’s new PHWs.^9^ This calculation is based on calculating the net difference in effect sizes between the two countries and multiplying by the number of smokers in a country, which in Zambia is 805 700.^49^ For example, the text-only health warning in Zambia led to a decrease of 1.5% of smokers who reported noticing the warnings ‘often/very often’. The introduction of the pictorial warnings in Kenya led to a 19.4% increase in noticing. The 20.9% net difference ×805 700 smokers in Zambia=168 392 smokers. Thus, the quasi-experimental analysis estimates that 168 392 ADDITIONAL smokers in Zambia would have noticed the warnings if Zambia had implemented PHWs like Kenya. Following the same formula for the other measures, if Zambia had implemented PHWs: 110 381 more Zambian smokers would have read the warnings; 133 746 more smokers would have thought about the health risks of smoking; 99 907 more smokers would have thought about quitting; 203 842 more smokers would have avoided the warnings—which, again, is a positive sign of warning impact^24 40–42^; and 85 404 more smokers would have given up a cigarette because of the warnings. These calculations demonstrate that the text-only warning in Zambia continues to be a lost opportunity for increasing the knowledge and awareness of the specific harms of cigarettes among the 805 700 smokers in Zambia as well as the many thousands of non-smoking Zambian youth, but who, with appropriate education (specifically from PHWs), could avoid ever initiating the use of cigarettes, which kill 7900 Zambians annually.^49^

An additional reason for the low impact of the single text-only warnings in Zambia may have been due to their small text size (less than 30%), which falls below the FCTC recommendation of at least 50% of both sides of the package. Furthermore, the use of an English-only text health warning in Zambia—a population with low literacy levels (Zambia’s English literacy level is 55% vs 82% in Kenya)—further limits its effectiveness. In contrast, in Kenya, the PHWs were large (30% front, 50% back) and the text was in both English and Kiswahili. ITC evidence from Uruguay has shown that increasing the size of existing PHWs leads to increases in their effectiveness.^11^ Thus, Kenya could benefit further from enhancing their PHWs beyond the minimum guidelines; there has not been a change in the size of warnings in Kenya since 2008. Furthermore, as of April 2021, Kenya had not yet implemented the remaining 12 PHWs included in the 2014 Tobacco Control Regulations; the same three PHWs have been on tobacco packages since September 2016. ITC studies have highlighted the importance of rotating and revising health warnings periodically (every 12–36 months) to reduce wear-out effects.^33 34^

### Strengths and limitations

Strengths of this study include the use of population-based longitudinal data across the pre- and post-policy implementation period and the use of consistent health warning-specific measures in each country at each wave, which included six validated indicators that have been used to evaluate the effectiveness of health warnings in 29 ITC countries, and are also listed in the 2008 International Agency of Research on Cancer Cancer Prevention Handbook, *Methods for Evaluating the Effectiveness of Tobacco Control Policies*.^39^ This consistency between waves and across countries strengthens the evaluation of HWI. Moreover, the health warning specific measures also sharpens the distinction between evaluating the effectiveness of health warnings on short-term (proximal) outcomes and on downstream future (distal) outcomes. Evaluating the impact of health warnings on those broad, downstream outcomes, such as quitting smoking behaviours, requires a substantial control of all confounding factors before estimating the effect of health warnings, which is nearly impossible. Health warning-specific measures, on the other hand, do not require a substantive control of other factors given that they are specifically linked to health warnings. This simplifies the estimation models and makes the estimation more reliable.

A limitation of this study is the timing of the follow-up surveys. The follow-up surveys of the two countries were not conducted at the same time and the Kenya follow-up survey was conducted almost 2 years after the implementation of the pictorial warnings. However, this delay may only be biased toward the null hypothesis of no substantive differences in effects between pictorial and text warnings.

There are also potential differences in interpretation of the questions given that multiple languages were involved in the surveys. That said, great care was taken in the translations of the questions from English to the local languages by fully bilingual translators. There may have been other differences in the tobacco control environments in both countries that we did not control for, such as educational campaigns or efforts besides the health warnings in each country that may have affected smokers’ responses. However, as stated above, given the evaluation measures in this study were specific to health warning impact, that specificity makes it unlikely that differences in those measures would have been affected by other tobacco control measures.

## Conclusions

This first quasi-experimental evaluation of the effectiveness of health warnings and the potential differences between text-only warnings and PHWs among smokers from two African countries demonstrated that the introduction of three PHWs on cigarette packs in Kenya led to a significant increase on all measures of HWI, while Zambia’s single text-only English warning showed a weak impact over a similar time period. Findings highlight and support current initiatives to introduce PHWs in Zambia and 33 other countries in the African Region that still have text-only health warnings, of which 31 are FCTC Parties and are thus obligated to implement PHWs.^50^

**What this paper adds**

- It is well established that pictorial health warnings (PHWs) are more effective than text-only warnings in communicating health risks of tobacco use.
- However, nearly all evidence regarding the effectiveness of PHWs has come from studies in high-income countries; few studies have been conducted in Africa, where the advantages of graphical warnings may be even greater, due to lower literacy levels which make text-only warnings less effective.
- This is the first quasi-experimental evaluation of PHWs in the African Region.
- This study demonstrates that the benefits of switching from text-only warnings to PHWs (eg, resulting in a significant increase in awareness of harms of tobacco use among smokers) are not limited to high-income Western countries but are also applicable to African countries like Kenya.
- Findings highlight and support current legislative initiatives to introduce PHWs in Zambia and 33 other African countries that still have text-only warnings, of which 31 are Parties of the Framework Convention on Tobacco Control and are thus obligated to implement PHWs.

## Data Availability

Data are available on reasonable request. Data from the International Tobacco Control Policy Evaluation (ITC) Project are available to approved researchers 2 years after the date of issuance of cleaned data sets by the ITC Data Management Centre. Researchers interested in using ITC data are required to apply for approval by submitting an International Tobacco Control Data Repository (ITCDR) request application and subsequently sign an ITCDR Data Usage Agreement. To avoid any real, potential or perceived conflict of interest between researchers using ITC data and tobacco-related entities, no ITCDR data will be provided directly or indirectly to any researcher, institution or consultant that is in current receipt of any grant monies or in-kind contribution from any tobacco manufacturer, distributor, or other tobacco-related entity. The criteria for data usage approval and the contents of the Data Usage Agreement are described online (http://www.itcproject.org).
